# Brain science and AI technology in the post-COVID era

**DOI:** 10.1093/nsr/nwab040

**Published:** 2021-03-18

**Authors:** Mu-ming Poo

As COVID-19 pandemic is gradually under control through large-scale vaccination, the world is moving into the post-COVID era that few societies are well-prepared for. Besides facing persistent threats imposed by SARS-CoV-2-related viruses and their variants, wide-ranging consequences of this pandemic will affect the mental health and education for large populations around the globe.

The infection of SARS-CoV-2 produces direct and indirect impacts on brain functions. Evidence is accumulating for neurological, cognitive and emotional deficits, as well as cerebrovascular disruptions in COVID-19 convalescent patients. These consequences of the infection need to be monitored over long periods, and potential underlying mechanisms, including direct viral infection of the brain, immune reaction against the virus, and other indirect effects need to be investigated.

Acute neurological symptoms of COVID-19 could result from direct invasion of SARS-CoV-2 into the nervous system through the olfactory bulb as well as nerve terminals and sensory receptors in the lungs and gastrointestinal tract. Overstimulation of the human immune system, exacerbated by the cytokine storm, could activate microglia and astrocytes in the brain, leading to neuroinflammatory responses and neurodegeneration. Neuroinflammation underlies many brain disorders and evidence is mounting for its link to the peripheral immune system. COVID-19 infection may induce *de novo* neurodegenerative diseases or aggravate pre-existing cognitive decline.

For both infected and uninfected individuals, the prolonged disease- and work-associated stress, social distancing, and quarantine may affect the mental health of large populations of all ages. Long-term neurological and psychiatric consequences of COVID-19 may include depression, insomnia, cognitive decline, and accelerated aging. The global population of reported cases of infection has now exceeded 100 million, and indirectly impacted population is much larger. Large-scale longitudinal assessment of the mental status of these populations remains outstanding. These are imminent post-COVID challenges to both the brain research community and medical care systems.

The use of digital technologies such as mobile apps, global positioning system,  social network, transaction data, and other personal information for contact tracing has contributed greatly to reduce the spread of COVID-19 infection. Based on large amounts of labeled clinical data, deep learning programs could assist clinical diagnosis and prognosis by identifying COVID-19 signatures in X-ray radiogram, CT images, and blood sample data that are distinct from those associated with tuberculosis and other respiratory disorders. These programs could also help to predict COVID infection based on symptoms, such as the loss of taste and smell, weakness, and characteristics of the cough sound, together with data on the age, sex and other personal characteristics. As auxiliary tools, AI methods will become invaluable in elevating the speed and accuracy of screening and managing infectious diseases in the post-COVID world.

Perhaps the most important long-term social impact of COVID-19 is the prominent role of online education. There is already a rapid growth of massive open online courses (MOOCs) around the globe prior to the pandemic. In China, the number of MOOCs has reached 5000, leading the world in both the number of participating schools and the students enrolled (over 70 million). In December 2019, the Bureau of Higher Education of China's Ministry of Education announced a ‘China MOOC Action Manifesto’, charting the blueprint of China MOOCs in the future, with the goals of achieving higher equity, quality, and availability across all regions in China, developing individualized and precise-targeting instruction, and devising mechanisms for resource sharing. These goals were echoed one year later in December of 2020, during a global conference sponsored by Tsinghua University in Beijing, where the Global MOOC Alliance was formed among 17 world-leading universities across 14 countries in all 6 continents.

The post-COVID online courses are likely to be further adapted to much larger student populations, with elevated efficiency and innovative use of the internet technologies, such as 5G network, virtual reality, and block chains. Novel course designs will allow immediate teacher-student and student-student interaction, provide home-based laboratory exercise and examination, as well as adaptive individualized instruction. Importantly, the efficient use of new AI technologies requires large-scale re-training of instructors. Obvious deficiencies associated with the lack of direct physical contact notwithstanding, the orientation towards network-based education is inevitable and will have profound consequences in education around the world, making high-quality education more accessible, particularly for resource-deficient regions of the world.

COVID-19 pandemic also brought into focus the fundamental issues of balancing personal freedom/privacy versus collective welfare, and national versus global interests. Brain science and AI are not only charting out new frontiers for human health care and technological advance, but also directly confronting these issues. While global consensus is difficult to achieve, due to ideological, cultural, religious and other factors, the pandemic experience has strongly highlighted the urgency and need for international consultation and collaboration in the Post-COVID era.

